# Genomic snapshot of *Klebsiella spp.* isolates from clinically ill animals reveal diverse lineages with limited relatedness to human isolates

**DOI:** 10.1186/s12917-025-04686-z

**Published:** 2025-07-11

**Authors:** Gordon Martin, Gregory H. Tyson, Jake Guag, Errol Strain, Olgica Ceric

**Affiliations:** 1 Center for Veterinary Medicine, United States Food and Drug Administration, Laurel, MD USA; 2 Veterinary Laboratory Investigation and Response Network (Vet-LIRN), Center for Veterinary Medicine, United States Food and Drug Administration, 8401 Muirkirk Rd, Laurel, MD 20708 USA

**Keywords:** *Klebsiella spp*, Antimicrobial resistance, Antimicrobial resistance monitoring, One health, Whole-genome sequencing

## Abstract

**Background:**

*Klebsiella* spp. is an important human and animal pathogen, and it is commonly found with resistance to clinically important antimicrobials worldwide. The main goals of this study were to determine the prevalence of antimicrobial resistance genes in our study population and to assess the relatedness between *Klebsiella* spp. isolated from humans and animals. Isolates were collected in 2019 and 2020 from various animal hosts that presented to veterinary hospitals in the U.S. that participate in the FDA’s Center for Veterinary Medicine Veterinary Laboratory Investigation and Response Network’s antimicrobial resistance monitoring program.

**Results:**

We sequenced a total of 204 *Klebsiella* spp. isolates. A majority of isolates were identified as *K. pneumoniae* (149/204, 73.0%), followed by *K. quasipneumoniae* (30/204, 14.7%), *K. variicola* (15/204, 7.4%), *K. aerogenes* (5/204, 2.5%), *K. oxytoca* (4/204, 2.0%), and *K. grimontii* (1/204, 0.5%). Out of 204 isolates, 138 were recovered from dogs, 25 from horses, 17 from cats, 6 from avian species, 5 from cows and 3 from pigs. The remaining 10 isolates were recovered from a few other mammal species. *Klebsiella* spp. isolates were very diverse. In silico multilocus sequence typing (MLST), using WGS data, identified a total of 88 known sequence types across all isolates. Seventeen isolates were not assigned an MLST sequence type due to combinations of alleles not previously found in the PubMLST database. 45 of the 204 isolates were assigned to 20 different single nucleotide polymorphism (SNP) clusters in the National Center for Biotechnology Information (NCBI) Pathogen Detection browser, and out of those, four isolates were assigned SNP clusters that also contained human isolates, all from dogs. The closest human isolate was 29 SNPs from a dog isolate. A total of 36 resistance genes were identified. The three most common resistance genes were *oqxAB*, *fosA*, and *bla*_SHV_. None of the isolates had carbapenem resistance genes, although one isolate from a goat had *mcr-8.1*, a colistin resistance gene.

**Conclusions:**

To our knowledge, this is the largest collection of sequenced *Klebsiella* from sick animals ever assembled, and the results found limited relatedness between these isolates and those from humans, despite the diversity of sequenced isolates.

**Supplementary Information:**

The online version contains supplementary material available at 10.1186/s12917-025-04686-z.

## Background

Antimicrobial resistance (AMR) is a global public health threat, with more than 35,000 people dying each year due to AMR in the United States [1]. AMR is also a One Health issue because AMR emergence in bacteria from humans, animals, or the environment can impact the health of the others [2]. AMR monitoring programs in the US have historically focused on tracking changes in antimicrobial susceptibility in bacteria from retail meats, food animals and sick humans to monitor changes in AMR over time [3]. It is essential that data from animal pathogens collected by veterinary diagnostic laboratories also be incorporated into AMR monitoring activities as part of the One Health framework [4]. These data, from bacterial pathogens of clinically ill veterinary patients, are an important addition to other large monitoring programs, such as the National Antimicrobial Resistance Monitoring System (NARMS). This is important because resistant pathogens in animals can result in zoonotic or foodborne illnesses in humans [5].

In March 2015, the first National Action Plan for Combating Antibiotic-Resistant Bacteria (CARB) was released [6] to guide the United States government, public health, healthcare, and veterinary partners in addressing AMR. In 2020, the second CARB plan was released [7]. The 2020 plan builds on the plan released in 2015 and presents coordinated, strategic actions that the United States Government will take in 2020–2025 by expanding evidence-based activities that have been shown to reduce AMR.

Since 2017, the Veterinary Laboratory Investigation and Response Network (Vet-LIRN), a program located within the FDA’s Center for Veterinary Medicine (CVM), has been conducting AMR monitoring of specific pathogens collected from sick animals to advance the CARB initiative [8]. These activities include antimicrobial susceptibility testing (AST) and whole-genome sequencing (WGS) of veterinary pathogens isolated at veterinary diagnostic laboratories. Vet-LIRN’s AMR monitoring program scope focuses on three pathogens: *Escherichia coli* and *Staphylococcus pseudintermedius* in dogs and *Salmonella enterica* in any animal host [8]. In addition, Vet-LIRN laboratories are conducting AST and WGS testing of other veterinary pathogens, including the *Klebsiella pneumoniae* complex. This work is in line with the CARB action plan and supports CVM’s comprehensive action to combat AMR. As of January 2023, Vet-LIRN collected AST data for more than 20,000 animal pathogen isolates, and more than 7,000 isolates were sequenced. The data provide a snapshot of the AMR profiles of the animal pathogens being isolated at veterinary diagnostic laboratories.

The members of the *K. pneumoniae* complex are increasingly important bacterial pathogens that can cause severe and life-threatening diseases such as urinary tract infections, pneumonia, enteritis, and bloodstream infection (septicemia) in both humans and animals [9–11]. The *K. pneumoniae* complex comprises *K. pneumoniae*, *K. quasipneumoniae* subspecies *quasipneumoniae*, *K. quasipneumoniae* subspecies *similipneumoniae*,* K. variicola* subspecies *variicola*, *K. variicola* subspecies *tropica*, K. *quasivariicola*, and *K. africana* [9, 12, 13]. Resistance in *K. pneumoniae* to last resort treatment (carbapenem antibiotics) has spread to all regions of the world and is a major cause of hospital-acquired infections in humans, such as pneumonia, bloodstream infections, and infections in newborns and intensive-care unit patients [14].

In addition to being a common hospital-acquired pathogen, *K. pneumoniae* has been recognized as a community-acquired pathogen in humans, commonly associated with bacteremia caused by urinary tract infection, vascular catheter infection, cholangitis, and enteritis [15]. In some countries (e.g., Iran, Georgia, Greece, India), carbapenem antibiotics do not work in more than half of the patients treated for *K. pneumoniae* infections due to antimicrobial resistance [14, 16]. Resistance to other antibiotics also varies considerably, as the rate of resistance to ciprofloxacin, an antibiotic commonly used to treat urinary tract infections, varied from 4.1 to 79.4% for *K. pneumoniae* [14] in countries reporting to the Global Antimicrobial Resistance and Use Surveillance System (GLASS). *K. quasipneumoniae* has also shown the capability to acquire resistance [17]. *Klebsiella variicola* is an opportunistic pathogen in humans where it is often antimicrobial-resistant [18], but it has not been thoroughly investigated in isolates from nonhuman sources. However, recent research confirmed the presence of *K. variicola* in the urine of two healthy heifers (young cows that had not yet had a calf), the first identification of *K. variicola* in the bovine urinary tract and the first confirmed *K. variicola* isolate encoding for flagella-mediated motility [19].

Multiple studies evaluated prevalence of *K. pneumoniae* antibiotic resistance genes (ARGs) in isolates from humans, animals, and environmental sources [20–22], with some studies reporting that up to 70% of the clinical isolates were found to be extended-spectrum beta-lactam (ESBL) producing *K.* pneumoniae, with *bla*_SHV_ being the most prevalent ARG (92.5%), followed by *bla*_CTX–M_ (67.5%) [21]. Plasmids have also been found with nearly identical sequences in *K. pneumoniae* from animals and humans [23], indicating the One Health nature of this pathogen. Plasmids have an important role in ARG spread within the One Health framework because the plasmid-mediated spread of ARGs can occur at different biological levels including between animal, human and environmental habitats [24]. Foods and food animals are also potential reservoirs of *K. pneumoniae* causing human illnesses [25]. As a result, it is important to understand the AMR of *K. pneumoniae* isolated from animals, as well as the relatedness to any bacteria causing illnesses in humans. Infections caused by *K. pneumoniae* also significantly impact animal health. As an opportunistic pathogen, *K. pneumoniae* has been implicated in cases of mastitis in cattle [26, 27], metritis in mares [28], bacteremia in calves [29], and pneumonia and urinary tract infections in dogs [9, 10, 30].

Here, we describe Vet-LIRN’s AMR monitoring program isolate collection and WGS testing results for 207 *Klebsiella* spp. isolates collected in 2019 and 2020 from various animal hosts that presented to veterinary hospitals in the U.S. Specifically, we sought to determine the prevalence of ARGs in our study population, as well as to assess the relatedness between *Klebsiella* spp. isolated from humans and animals.

## Results

### *Klebsiella* spp. And animal host/isolation sources

Initial identification of the 207 *Klebsiella* spp. isolates, using either analytical profile index (API), matrix assisted laser desorption/ionization time of flight (MALDI-TOF) mass spectrometry, polymerase chain reaction (PCR), Sensititre, Vitek, or biochemical assay, identified three *K. aerogenes*, four *K. oxytoca*, 198 *K. pneumoniae*, one *K. variicola*, and one with only a genus level *Klebsiella* identification. WGS later confirmed a total of 204 isolates as *Klebsiella* spp. and only these 204 isolates were included in the final analysis. According to WGS, the most common species belonged to the *Klebsiella pneumoniae* complex, *K. pneumoniae* (149/204, 73.0%), followed by *K. quasipneumoniae* (30/204, 14.7%) and *K. variicola* (15/204, 7.4%), along with other *Klebsiella* spp., *K. aerogenes* (5/204, 2.5%), *K. oxytoca* (4/204, 2.0%), and *K. grimontii* (1/204 0.5%).

Individual animal hosts and sample collection sites were combined into common groups for ease of visualization and comparison. Closely related animal host species were grouped together, and sample collection sites were grouped by body systems/organs. A complete list of common groups and respective individual animal hosts and collection sites of each *Klebsiella* spp. is contained in Supplemental File 1. Canines were the most common animal host (138/204, 67.6%), followed by equine (25/204, 12.3%), feline (17/204, 8.3%), other mammals (10/204, 4.9%), avian (6/204, 2.9%), bovine (5/204, 2.5%), and porcine (3/204, 1.5%). Interestingly, even though two-thirds of the animal hosts in this study were from canines, each of the four *K. oxytoca* isolates came from a unique animal host, canine, equine, feline, and another mammal, a deer, while four of the five (80%) *K. aerogenes* and 14 of the 15 (93.3%) *K. variicola* were isolated from canines (Supplemental File 1). *Klebsiella* isolates were collected from a variety of sources, with the urinary tract being the most common body system (94/204, 46.1%), comprising just under half of the isolates, followed by the skin (41/204, 20.1%) and respiratory tract (35/204, 17.2%). Two of the five *K. aerogenes* isolates were collected from the urinary tracts of dogs, the sole *K. grimontii* from bovine skin, while all four *K. oxytoca* isolates were isolated from different animal hosts and body systems/organs (Supplemental File 1).

### Spatial distribution of *Klebsiella* spp. Isolates

**Fig. 1:**
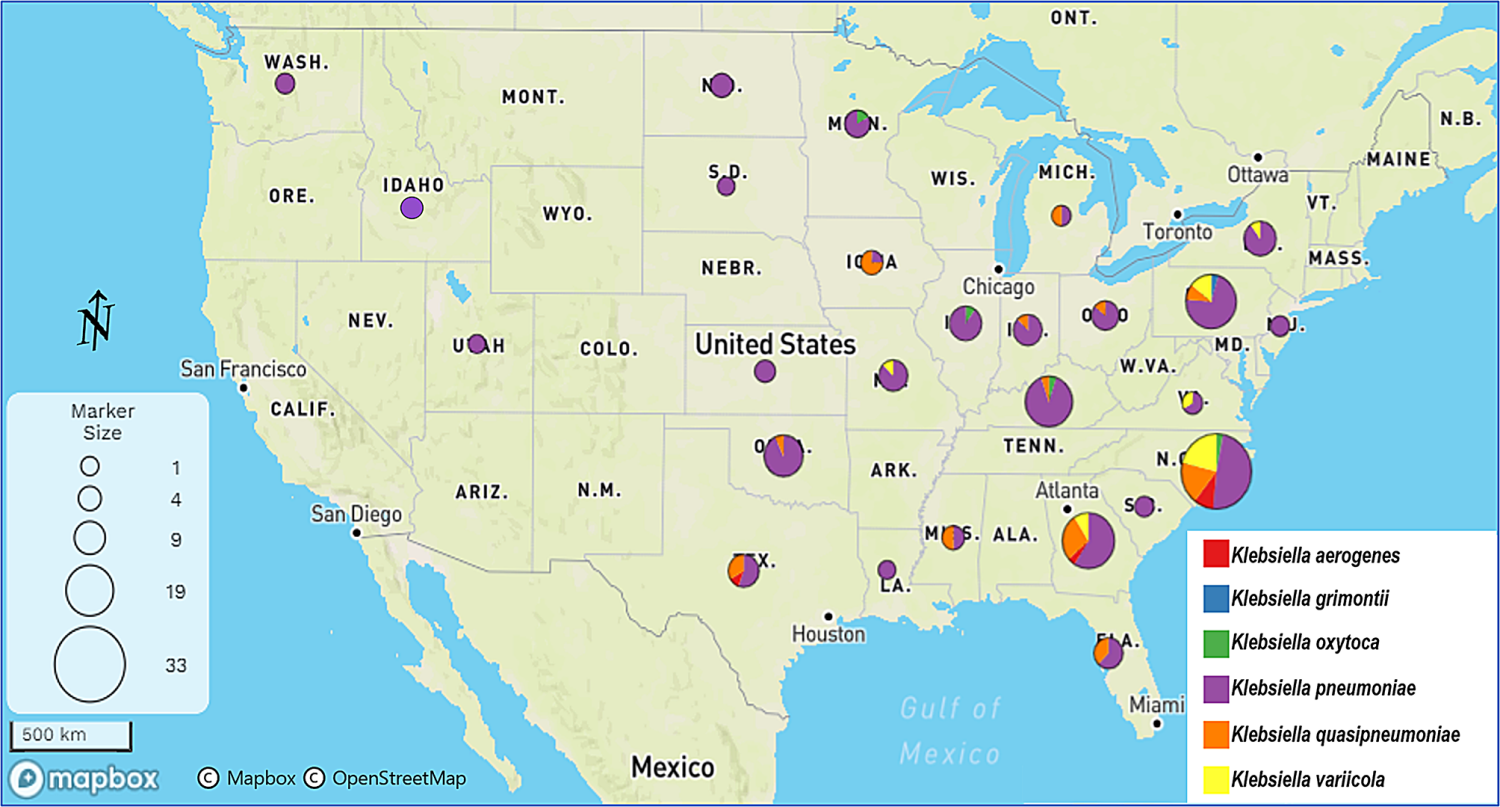
Spatial distribution of *Klebsiella* spp. isolates. The map represents the geographic distribution of all *Klebsiella* spp. isolates from this study collected between January 2019 and December 2020. The pie charts are proportionally sized according to how many isolates of a given species were collected from each state. Pie chart sizes and their corresponding number of isolates are shown in the “Marker Size” legend

Isolates were collected from Vet-LIRN labs located mostly in the central and eastern regions of the United States (Fig. 1). The highest number of isolates was collected from North Carolina (33/204), which also demonstrated the highest amount of species diversity (5/6 of the total species collected), followed by Pennsylvania (4/6 species) and Georgia (4/6 species) (Fig. 1).

All five *K. aerogenes* isolates were collected from three southern states, one from Georgia, three from North Carolina, and one from Texas, while each of the four *K. oxytoca* isolates were collected from a different state, North Carolina, Illinois, Kentucky, and Minnesota. The sole *K. grimontii* isolate was collected from Pennsylvania.

### Isolate diversity and relatedness

**Fig. 2:**
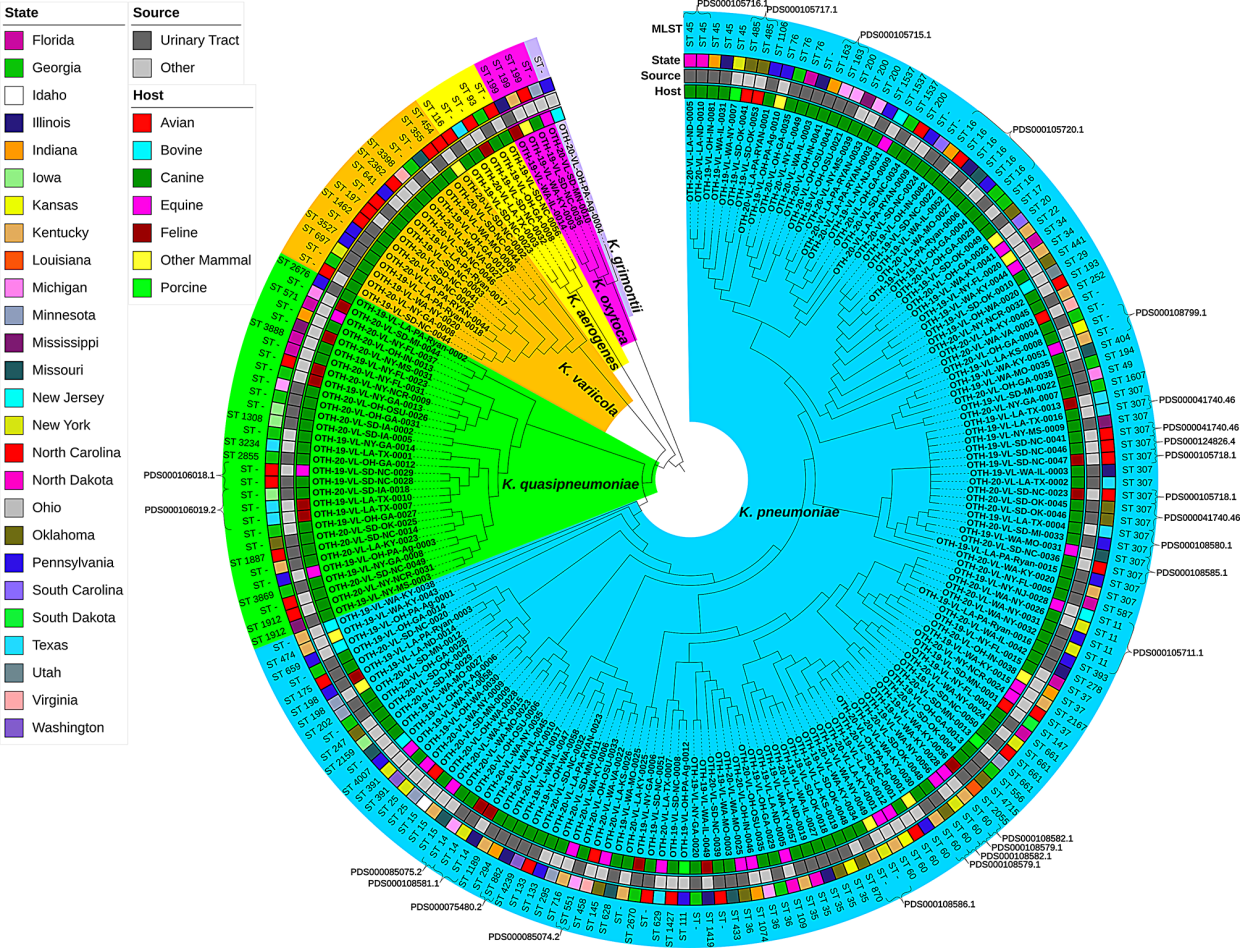
Cladogram of 204 *Klebsiella* spp. Reference-free maximum-likelihood cladogram of 204 Klebsiella spp. isolates, core genes were aligned using kSNP4 and visualized using The Interactive Tree of Life (iTOL). The color rings, starting with the innermost and moving outward, denote the *Klebsiella* species, animal host, sample collection source (urinary tract or “other”), and the state the sample was collected from

A core-genome SNP analysis was used to construct a cladogram to help visually assess the genetic relatedness of isolates and simultaneously view associated metadata: species, animal host, isolation source, and MLST sequence type (Fig. 2). A detailed table of NCBI SNP Clusters assigned at the time of this writing and associated isolate metadata and totals are available in Supplemental File 2.

**Fig. 3:**
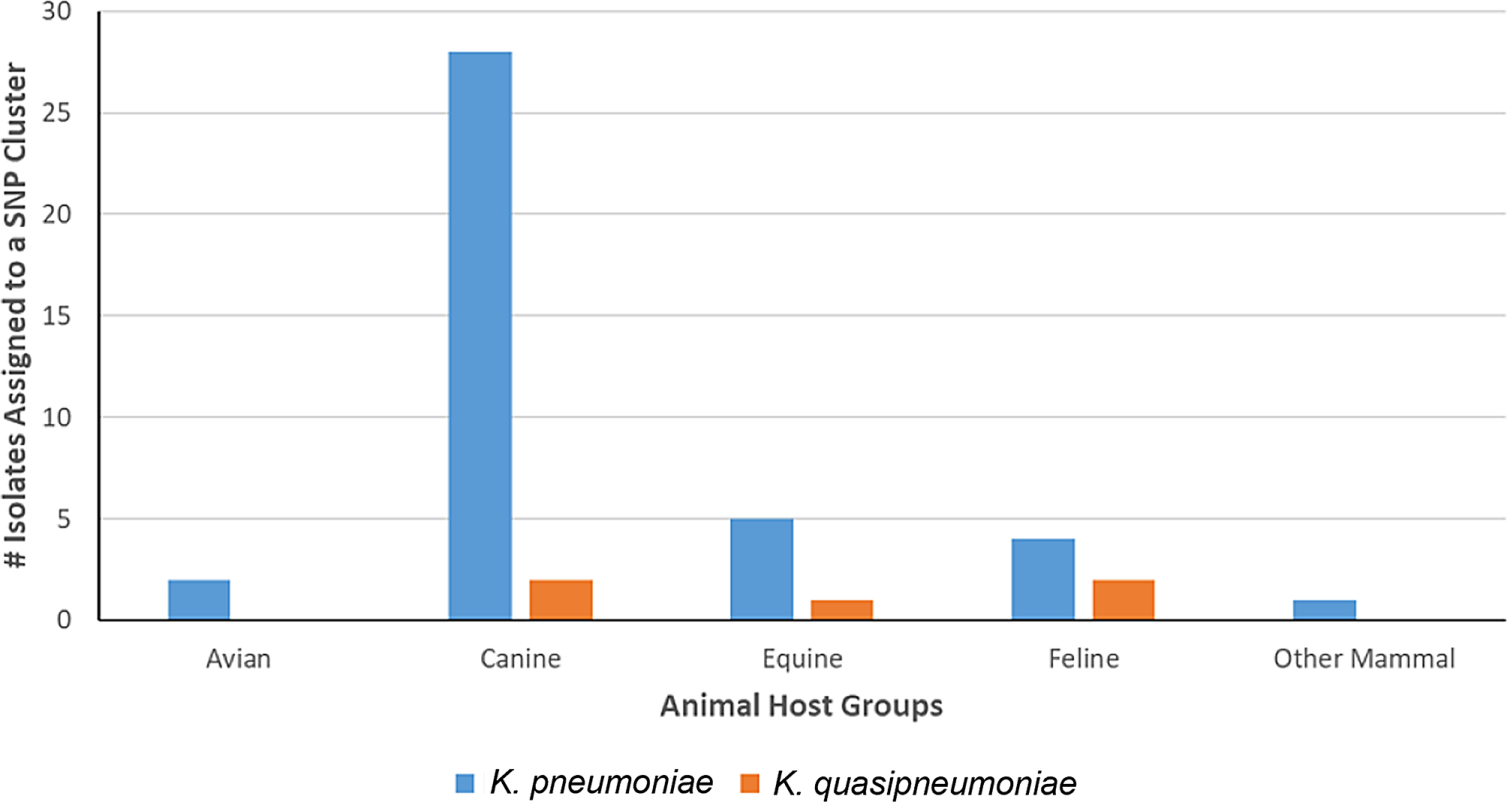
Breakdown of the forty-five *Klebsiella pneumoniae complex* isolates assigned to an NCBI Pathogen Detection SNPs Cluster and associated common animal host group. The X-axis represents animal host groups. The Y-axis represents the number of isolates from each animal host assigned to an NCBI SNP cluster

The isolates in this study were diverse. 45 of the 204 isolates (22%) were assigned to 20 different SNP clusters in the NCBI Pathogen Detection browser (Fig. 2 and Supplemental File 2), indicating that the remaining isolates were not within 50 SNPs of any other publicly available sequences. All 45 isolates belonged to the *Klebsiella pneumoniae* complex, *K. pneumoniae* (40/45, 88.9%) and *K. quasipneumoniae* (5/45, 11.1%), with the majority isolated from dogs (30/45, 66.7%), in line with the overall percentage from canine sources. A majority (28/40, 70%) of *K. pneumoniae* isolates were collected from canines, but of the five *K. quasipneumoniae* isolates assigned to a SNP cluster, two were isolated from canines, two from felines, and one from an equine (Figs. 2 and 3).

Eight of the 20 SNP clusters assigned to isolates from this study contained isolates collected from multiple animal hosts. Isolates belonging to six of these eight SNP clusters were collected from both canines and equines, one SNP Cluster had isolates collected from canines and felines, and one SNP cluster had isolates collected from canines and “other mammals” (bear) (Fig. 2 and Supplemental File 2). Only five isolates were assigned to SNP clusters that also contained human isolates, all from dogs and belonging to MLST sequence type ST307 (OTH-19-VL-LA-TX-0016, OTH-19-VL-SD-NC-0041, OTH-20-VL-SD-OK-0045, OTH-20-VL-SD-OK-0046, and OTH-19-VL-SD-NC-0046), with four belonging to the same SNP cluster. Five NCBI SNP Clusters fell within MLST sequence type ST307, the most of all the sequence types (Fig. 2 and Supplemental File 2). The closest human isolates were 24 and 29 SNPs from a dog isolate, OTH-19-VL-LA-TX-0016 and OTH-19-VL-SD-NC-0041, respectively (Supplemental File 2).

**Fig. 4:**
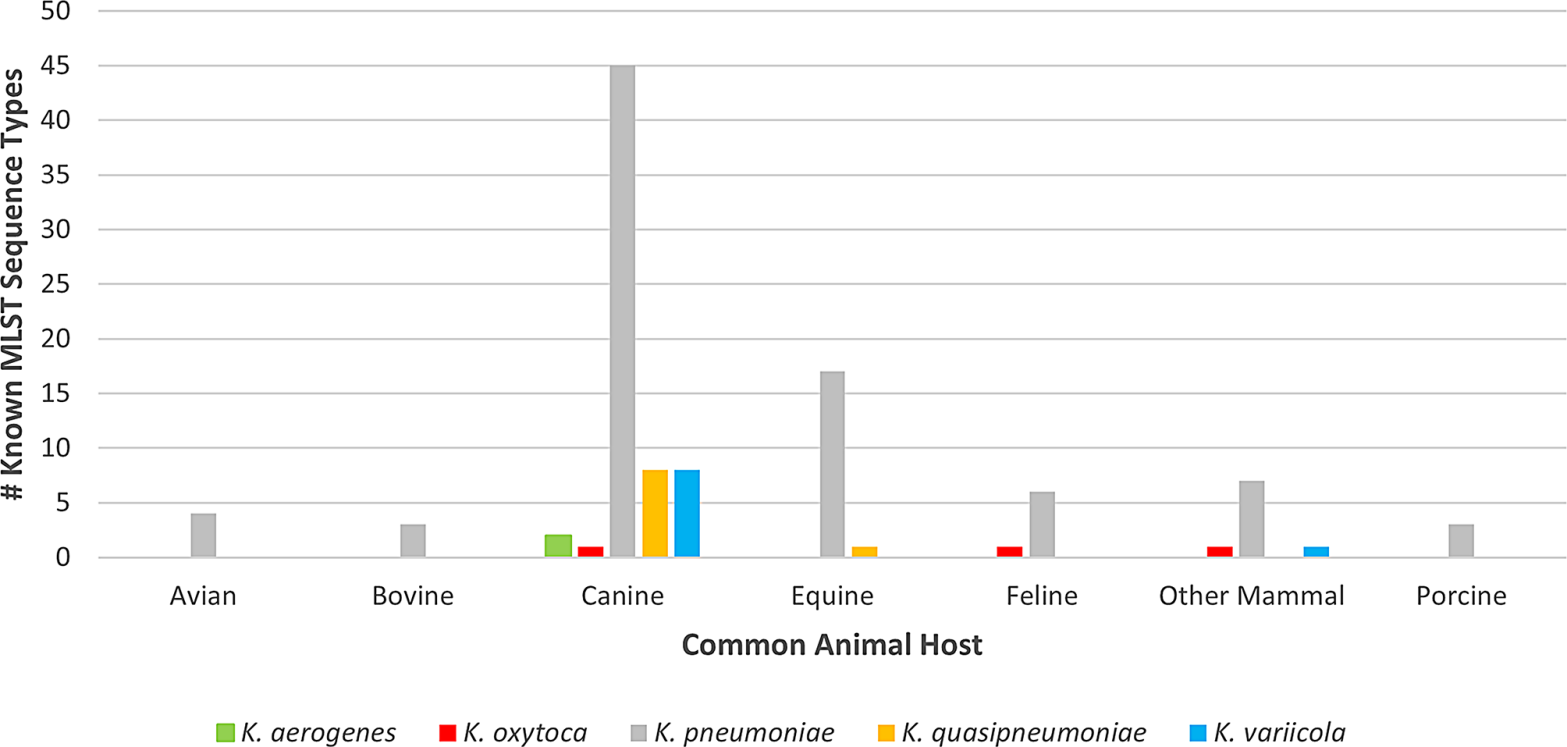
Known MLST STs by common animal host groups. The X-axis represents animal host groups. The Y-axis represents the number of known MLST sequence types within that category of animal host group and does not include unknown sequence types.

Multilocus sequence typing (MLST), using whole-genome sequencing data, identified 88 known sequence types (STs) across all 204 isolates in this study, with the majority (85/88, 96.6%) belonging to *Klebsiella pneumoniae* complex isolates (Fig. 2 and see Supplemental File 3 for a complete list of all the MLST sequence types and associated *Klebsiella* spp. and animal hosts). Sixty-seven known STs were identified among 132 of the 149 *K. pneumoniae* isolates. The largest number of known STs (*n* = 45) was found in *K. pneumoniae* canine isolates (85 out of 98 isolates), probably due to the large canine sample size (Figs. 2 and 4).

Similar to the NCBI Pathogen Detection SNP cluster assignments, this demonstrates the high degree of diversity among these isolates. Ten known MLST STs, all belonging to *K. pneumoniae*, were identified in 16 of the 20 total SNP clusters: ST11, ST14, ST16, ST163, ST307, ST45, ST485, ST551, ST60, and ST882. ST307 was the most common being found in 17 isolates (Fig. 2 and Supplemental File 3), with 11 of these being assigned to an NCBI SNP cluster (Fig. 2 and Supplemental File 2). The remaining 17 out of the 149 *K. pneumoniae* isolates were not assigned an MLST sequence type due to combinations of alleles not previously found in the PubMLST database. Ten of the 30 *K. quasipneumoniae* isolates belonged to nine known sequence types, eight from canines and one from an equine. Nine of the 15 *K. variicola* isolates belonged to nine known sequence types, all from canines. Two of the five *K. aerogenes* isolates each belonged to a unique known sequence type, ST93 and ST116, both from canines. Three of the four *K. oxytoca* isolates belonged to a single sequence type, ST199, but each was from a different animal host: canine, feline, and deer. The single *K. grimontii* isolate belonged to an unknown sequence type.

64 known STs were isolated from 138 canines. The most frequent sequence type in dogs was ST307 (13/138, 9.4%), followed by ST16 (7/138, 5.1%). ST35 was found in five isolates (5/138, 3. 6%), ST200 and ST45 were found in four isolates (4/138, 2,9%). ST661, ST11 and ST60 were found in three isolates, (3/138, 2.1%). Two isolates were ST15 and ST36 (138/2, 1.2%). The remaining STs were more sporadic, and most of them were identified on only one occasion (singletons), including ST14 (1/138, 0.7%). However, considering the total number of isolates collected from each animal host, canines were not necessarily proportionally the most diverse. Of the three pig isolates, all three had different STs, and one, ST-111, was unique only to that pig isolate. The six avian isolates had four different known STs and one unidentified STs. Of the four known STs, three, ST147, ST458, and ST485, were unique to avian isolates in our study. The eight *K. pneumoniae* collected from other mammals contained seven known STs and an unidentified sequence type, with two being unique only to the other mammals in our study (ST20 and ST474). For *K. pneumoniae*, the most common sequence type found was ST307 in 17 isolates. ST307 isolates represented diverse animal hosts, including thirteen dogs (149/13, 8.7%), three cats (149/3, 2%), and a horse isolate (149/1, 0.7%).

### Prevalence of resistance genes

A total of 36 resistance genes and three resistance-associated mutations were identified and their prevalence is shown in Table 1. The three most common resistance genes were *oqx*AB (201/204, 98.5%), which can contribute to quinolone and phenicol resistance; *fos*A (199/204, 97.5%), which encodes fosfomycin resistance; and *bla*_SHV_ (145/204, 71.1%), which confers beta-lactam resistance. Of the three resistance-associated mutations observed in this study, gyrA, conferring fluoroquinolone resistance, was the most common (36/204, 17.6%), followed by pmrB (20/204, 9.8%) and pmrA (1/204, 0.5%), both associated with colistin resistance.

**Table 1:** Antimicrobial class, resistance genes, and gene prevalence

Class	Gene	*N* (Prevalence)
AMINOGLYCOSIDE	*aac(3)*	40 (19.6%)
	*aac(6’)*	5 (2.5%)
	*aadA*	28 (13.7%)
	*ant(2’’)*	1 (0.5%)
	*aph(3’)*	47 (23.0%)
	*aph(6)*	43 (21.1%)
	*rmtB1*	1 (0.5%)
**AMINOGLYCOSIDE Total**		**59 (28.9%)**
AMINOGLYCOSIDE/QUINOLONE	*aac(6’)-Ib-cr*	37 (18.1%)
**AMINOGLYCOSIDE/QUINOLONE Total**		**37 (18.1%)**
BETA-LACTAM	*ampC*	5 (2.5%)
	*blaCMY*	10 (4.9%)
	*blaCTX-M*	31 (15.2%)
	*blaDHA*	5 (2.5%)
	*blaLAP*	7 (3.4%)
	*blaLEN*	14 (6.9%)
	*blaOKP*	30 (14.7%)
	*blaOXA*	30 (14.7%)
	*blaOXY*	5 (2.5%)
	*blaSHV*	145 (71.1%)
	*blaTEM*	45 (22.1%)
**BETA-LACTAM Total**		**202 (99.0%)**
COLISTIN	*mcr*	1 (0.5%)
	*pmrA* mutation	1 (0.5%)
	*pmrB* mutation	20 (9.8%)
**COLISTIN Total**		**22 (10.8%)**
FOSFOMYCIN	*fosA*	199 (97.5%)
**FOSFOMYCIN Total**		**199 (97.5%)**
MACROLIDE	*ere(A)*	2 (1.0%)
	*erm(B)*	1 (0.5%)
	*mph(A)*	16 (7.8%)
**MACROLIDE Total**		**17 (8.3%)**
PHENICOL	*catAB*	40 (19.6%)
	*cmlA*	1 (0.5%)
	*floR*	6 (2.9%)
**PHENICOL Total**		**43 (21.1%)**
PHENICOL/QUINOLONE	*oqxAB*	201 (98.5%)
**PHENICOL/QUINOLONE Total**		**201 (98.5%)**
QUINOLONE	*gyrA* mutation	36 (17.6%)
	*qnr*	41 (20.1%)
**QUINOLONE Total**		**52 (25.5%)**
RIFAMYCIN	*arr*	15 (7.4%)
**RIFAMYCIN Total**		**15 (7.4%)**
SULFONAMIDE	*sul*	58 (28.4%)
**SULFONAMIDE Total**		**58 (28.4%)**
TETRACYCLINE	*tetA*	42 (20.6%)
	*tetB*	1 (0.5%)
	*tetC*	1 (0.5%)
	*tetD*	12 (5.9%)
**TETRACYCLINE Total**		**55 (27.0%)**
TRIMETHOPRIM	*dfrA*	57 (27.9%)
**TRIMETHOPRIM Total**		**57 (27.9%)**
Isolate Total		204 (100.0%)

N = Number of isolates and (%) = Prevalence

Some notable findings include the common identification of beta-lactam resistance genes, including *bla*_SHV_ and *bla*_CTX−M_, and a plasmid-mediated *amp*C beta-lactamase gene, *bla*_DHA−1_. No carbapenem resistance genes were identified. 144 of the 145 (144/145, 99.3%) *bla*_SHV_ containing isolates were found in *K. pneumoniae* and only one (1/144, 0.1%) was found in a *K. variicola* (Supplemental File 4) isolate. 30 of the 31 (30/31, 96.8%) *bla*_CTX−M_ containing isolates belonged to *K. pneumoniae* and the remaining isolate (1/31, 3.2%) belonged to *K. oxytoca*.

Resistance mechanisms to fluoroquinolones were also prevalent, including 36 isolates with *gyr*A mutations (36/204, 17.6%) and 41 isolates with *qnr* gene acquisitions (41/204, 20.1%) (Supplemental File 4). The *qnr* genes are of particular concern since they can be transmitted to other previously susceptible isolates via plasmids. Notably, while *oqx*AB was found in nearly all isolates, this efflux pump rarely confers resistance on its own, instead acting synergistically with other resistance mechanisms.

Additional notable finding includes the identification of the mobile colistin resistance gene (*mcr*), *mcr-8.1*, isolated from the respiratory tract of a goat (Supplemental File 4). While the presence of *pmr*A and *pmr*B mutations in our isolate collection can also confer colistin resistance, these mechanisms are not transmissible.

**Fig. 5:**
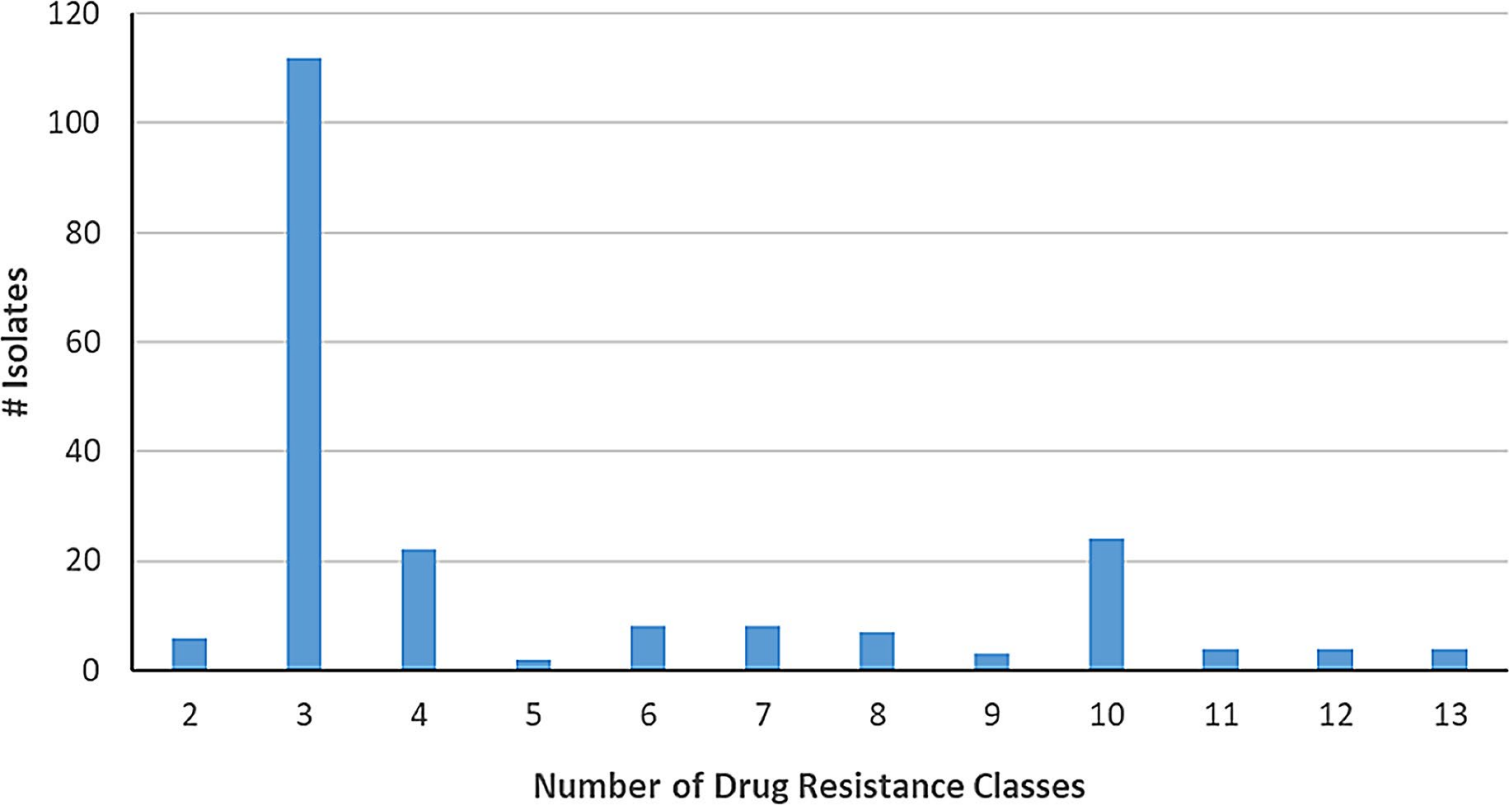
Prevalence of the number of resistance classes per isolate across all isolates. The X-axis represents the count of how many unique drug resistance classes were found in each isolate and does not include when the same drug class is found more than one in an isolate even if it is identified from a different gene or allele. The Y-axis represents the number of isolates.

Multidrug resistance was common among all isolates. The most common was resistance to *three* drug classes (112/204, 54.9%), followed by ten drug classes (24/204, 11.8%). The highest number of resistance classes in an isolate was 13 (4/204, 2.0%), and all isolates had resistance mechanisms for at least two drug classes (Fig. 5 and Supplemental File 5).

**Fig. 6:**
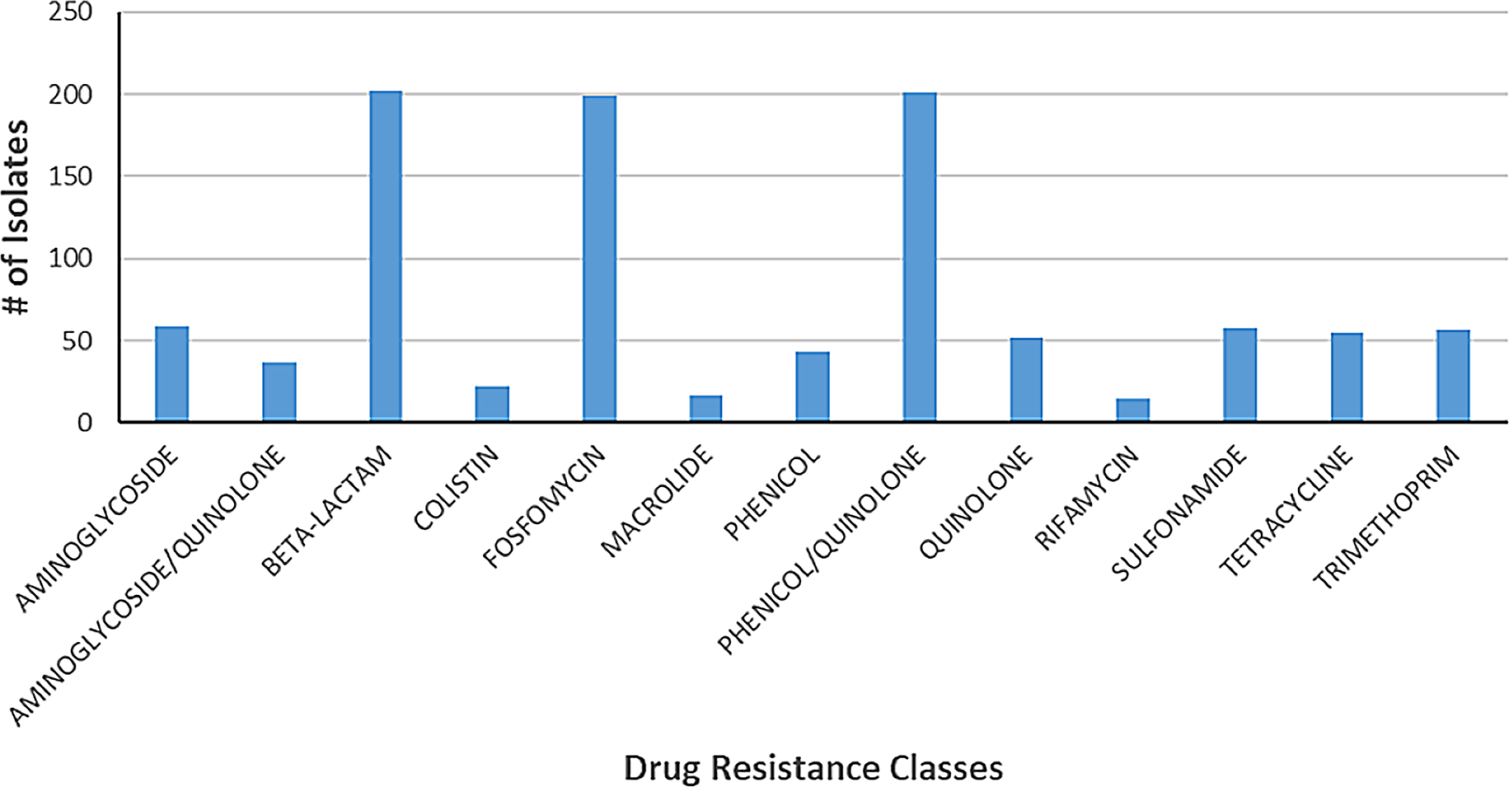
Prevalence of drug resistance classes. The X-axis shows all the drug resistance classes found in the study isolates. The Y-axis represents the number of isolates containing resistance genes or mutations for each drug class.

The most common resistance classes were beta-lactams (202/204, 99.0%), with bla_SHV_ genes being most common (145/204, 71.2%), phenicol/quinolone resistance due to *oqx*AB genes (98.5%), and fosfomycin resistance due to *fos*A genes(97.5%) (Fig. 6).

**Fig. 7:**
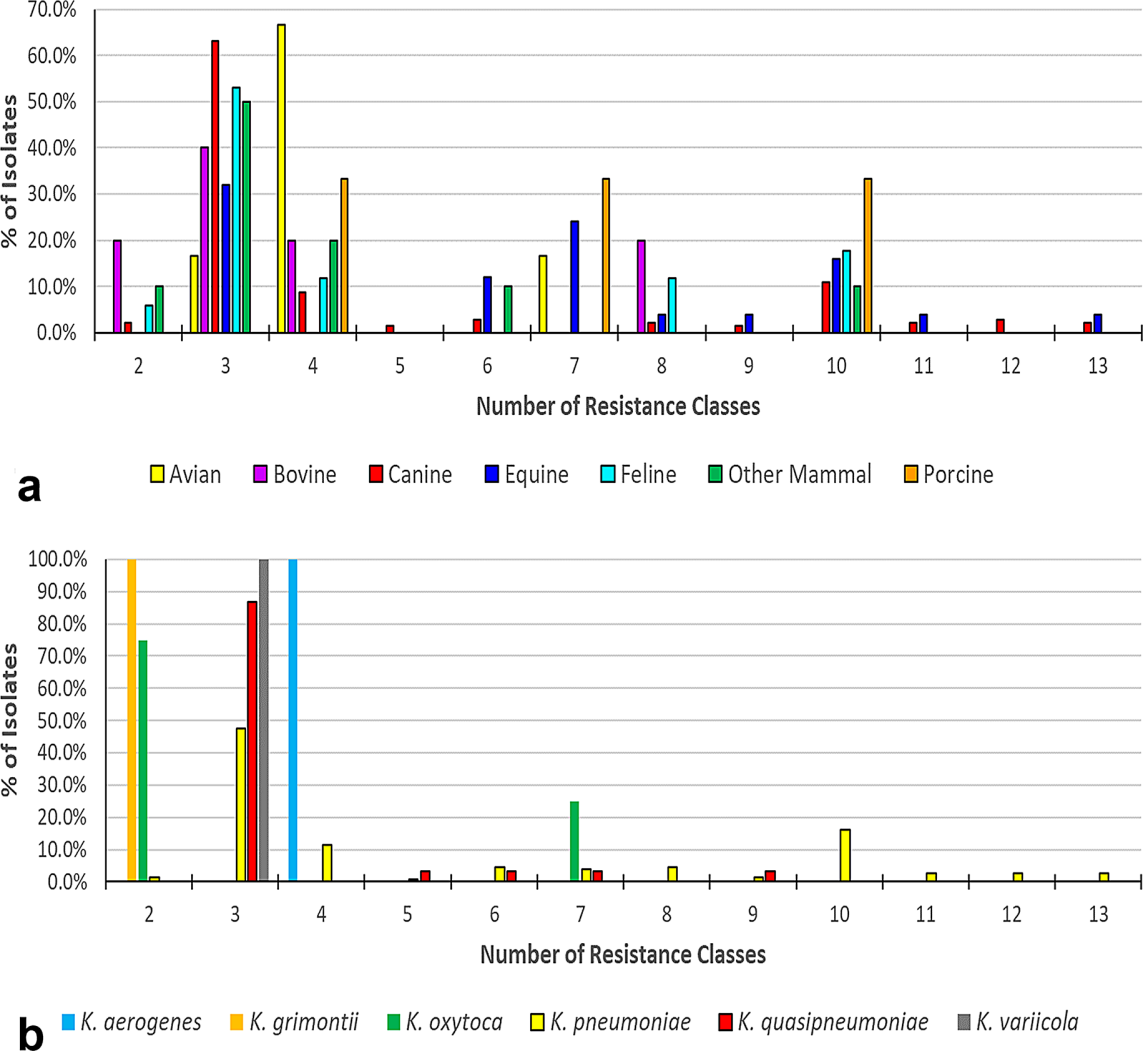
Number of resistance classes by common animal host group and *Klebsiella* spp. **a**: Number of resistance classes by common animal host group. The X-axis shows the number of unique drug resistance classes present in each isolate. The Y axis represents the prevalence of the number of drug resistance classes found within each animal host, not the overall prevalence across all isolates. **b**: Number of drug resistance classes by Klebsiella spp. The X-axis shows the number of unique drug resistance classes present in each isolate. The Y-axis represents the prevalence of the number of drug resistance classes found within each Klebsiella spp., not the overall prevalence across all isolates

Across all *Klebsiella* spp. from this study, canines and equines contained isolates with the highest number of resistance classes per isolate (Fig. 7a). The *bla*_DHA−1_ gene was identified in five isolates, all belonging to the *Klebsiella pneumoniae* complex, four *K. pneumoniae* (three canines and one equine) and one *K. quasipneumoniae* (canine) (Supplemental File 4). All four *bla*_DHA−1_ -harboring *K. pneumoniae* belonged to MLST sequence type ST11. *K. pneumoniae* was resistant to the most drug classes, with four isolates resistant to 13 drug classes, followed by *K. quasipneumoniae*, with one isolate resistant to nine drug classes, and one *K. oxytoca* isolate resistant to seven drug classes (Fig. 7b**).***K. aerogenes*,* K. grimontii*,* and K. variicola* were all resistant to 4 drug classes or less. All five *K. aerogenes* shared identical resistance profiles and were resistant to four drug classes: beta-lactams (*amp*C), fosfomycin (*fos*A), quinolones (*gyr*A(S83T) mutation) and phenicol/quinolone (*oqx*A). The sole *K. grimontii* isolate was resistant to two drug classes: beta-lactams (*bla*_OXY_) and phenicol/quinolone (*oqx*A). Three of the four *K. oxytoca* isolates shared identical resistance profiles and were resistant to two drug classes: beta-lactams (*bla*OXY) and phenicol/quinolone (*oqx*A). One *K. oxytoca* isolate was resistant to seven drug classes. *K. pneumoniae* and *K. quasipneumoniae* isolates demonstrated the highest range of antimicrobial resistance. *K. pneumoniae* resistance ranged from two to thirteen drug classes. Four *K. pneumoniae* isolates were resistant to 13 drug classes, three from canines and one from an equine, all had the same MLST sequence type (ST11) and had identical resistance profiles. *K. quasipneumoniae* resistance ranged from two to seven drug classes. All 15 *K. variicola* isolates had identical resistance drug class profiles, resistant to three classes: beta-lactams, fosfomycin, and phenicol/quinolone, and almost identical resistance gene and allele profiles. All 15 had identical resistance alleles for fosfomycin (*fos*A) and phenicol/quinolone (*oqx*AB). The only difference was that one *K. variicola* isolate had a *bla*_SHV_ beta-lactam gene (*bla*_SHV−1_), while the other 14 had identical beta-lactam genes (*bla*_LEN_) with some variations at the allele level.

### Plasmid analysis

Thirty-three distinct plasmid replicon types were found in 111 isolates (54.4%) from 12 of the 14 animal hosts (85.7%) (Table 2).

**Table 2:** Distribution of the 111 plasmid containing isolates across all *Klebsiella* spp. And animal host groups

	K. pneumoniae (*n* = 98)							K. quasipneumoniae (*n* = 10)			K. variicola (*n* = 3)		Row Totals
Plasmid Replicon	Avian (*n* = 5)	Bovine (*n* = 3)	Canine (*n* = 59)	Equine (*n* = 16)	Feline (*n* = 7)	Other Mammal (*n* = 5)	Porcine (*n* = 3)	Canine (*n* = 5)	Equine (*n* = 2)	Feline (*n* = 3)	Canine (*n* = 2)	Other Mammal (*n* = 1)	
Col(BS512)							1 (0.9%)						1 (0.9%)
Col(IMGS31)			2 (1.8%)										2 (1.8%)
Col156				1 (0.9%)									1 (0.9%)
Col440I			1 (0.9%)										1 (0.9%)
Col440II			1 (0.9%)	1 (0.9%)	2 (1.8%)								4 (3.6%)
ColpVC			1 (0.9%)	1 (0.9%)									2 (1.8%)
ColRNAI			3 (2.7%)										3 (2.7%)
FIA(pBK30683)				1 (0.9%)							1 (0.9%)		2 (1.8%)
IncA/C2		1 (0.9%)	2 (1.8%)										3 (2.7%)
IncFIA			1 (0.9%)										1 (0.9%)
IncFIA(HI1)			3 (2.7%)	2 (1.8%)		1 (0.9%)			1 (0.9%)		1 (0.9%)		8 (7.2%)
IncFIB (AP001918)	1 (0.9%)		1 (0.9%)										2 (1.8%)
IncFIB(K)	1 (0.9%)	2 (1.8%)	40 (36.0%)	7 (6.3%)	5 (4.5%)	2 (1.8%)	3 (2.7%)	1 (0.9%)				1 (0.9%)	62 (55.9%)
IncFIB(Mar)	1 (0.9%)	1 (0.9%)		1 (0.9%)	2 (1.8%)	1 (0.9%)						1 (0.9%)	7 (6.3%)
IncFIB (pKPHS1)			3 (2.7%)					1 (0.9%)					4 (3.6%)
IncFIB(pQil)										1 (0.9%)			1 (0.9%)
IncFII			3 (2.7%)			1 (0.9%)						1 (0.9%)	5 (4.5%)
IncFII(29)				1 (0.9%)									1 (0.9%)
IncFII(K)	1 (0.9%)		9 (8.1%)	1 (0.9%)	2 (1.8%)	2 (1.8%)	1 (0.9%)	2 (1.8%)		2 (1.8%)			20 (18.1%)
IncFII(pHN7A8)					1 (0.9%)								1 (0.9%)
IncHI1A(CIT)			1 (0.9%)										1 (0.9%)
IncHI1B		2 (1.8%)	1 (0.9%)	3 (2.7%)	2 (1.8%)	1 (0.9%)							9 (8.1%)
IncHI1B(CIT)			1 (0.9%)										1 (0.9%)
IncHI2				1 (0.9%)			1 (0.9%)						2 (1.8%)
IncHI2A				1 (0.9%)			1 (0.9%)						2 (1.8%)
IncI1			2 (1.8%)				1 (0.9%)						3 (2.7%)
IncI2				1 (0.9%)									1 (0.9%)
IncL/M (pMU407)			1 (0.9%)										1 (0.9%)
IncN				3 (2.7%)					1 (0.9%)				4 (3.6%)
IncQ1			3 (2.7%)	1 (0.9%)									4 (3.6%)
IncR	1 (0.9%)		9 (8.1%)	6 (5.4%)	2 (1.8%)	1 (0.9%)	1 (0.9%)	1 (0.9%)					21 (19.9%)
IncX1								1 (0.9%)					1 (0.9%)
IncX4			1 (0.9%)										1 (0.9%)

Only isolates belonging to the *Klebsiella pneumoniae* complex (*K. pneumoniae*,* K. quasipneumoniae*, and *K. variicola)* were found to contain plasmids in this study. Of the 111 plasmid-containing isolates, 98 (88.3%) were *K. pneumoniae*, with 31 of the 33 replicons present, 22 of which were exclusive to *K. pneumoniae*. Ten of the 111 isolates (9.0%) were *K. quasipneumoniae* and contained 11 replicons, two of which were found only in *K. quasipneumoniae*. The remaining three isolates (2.7%) were *K. variicola* and had five replicons, none of which were exclusive to *K. variicola*. IncFIB(K) was the most common plasmid observed in *K. pneumoniae*, IncFII(K) in *K. quasipneumoniae*, while the three *K. variicola* plasmid-containing isolates harbored five distinct plasmid replicons. One isolate, *K. pneumoniae* from the respiratory tract of a horse, contained five distinct plasmid replicons, the most in this study.

**Fig. 8:**
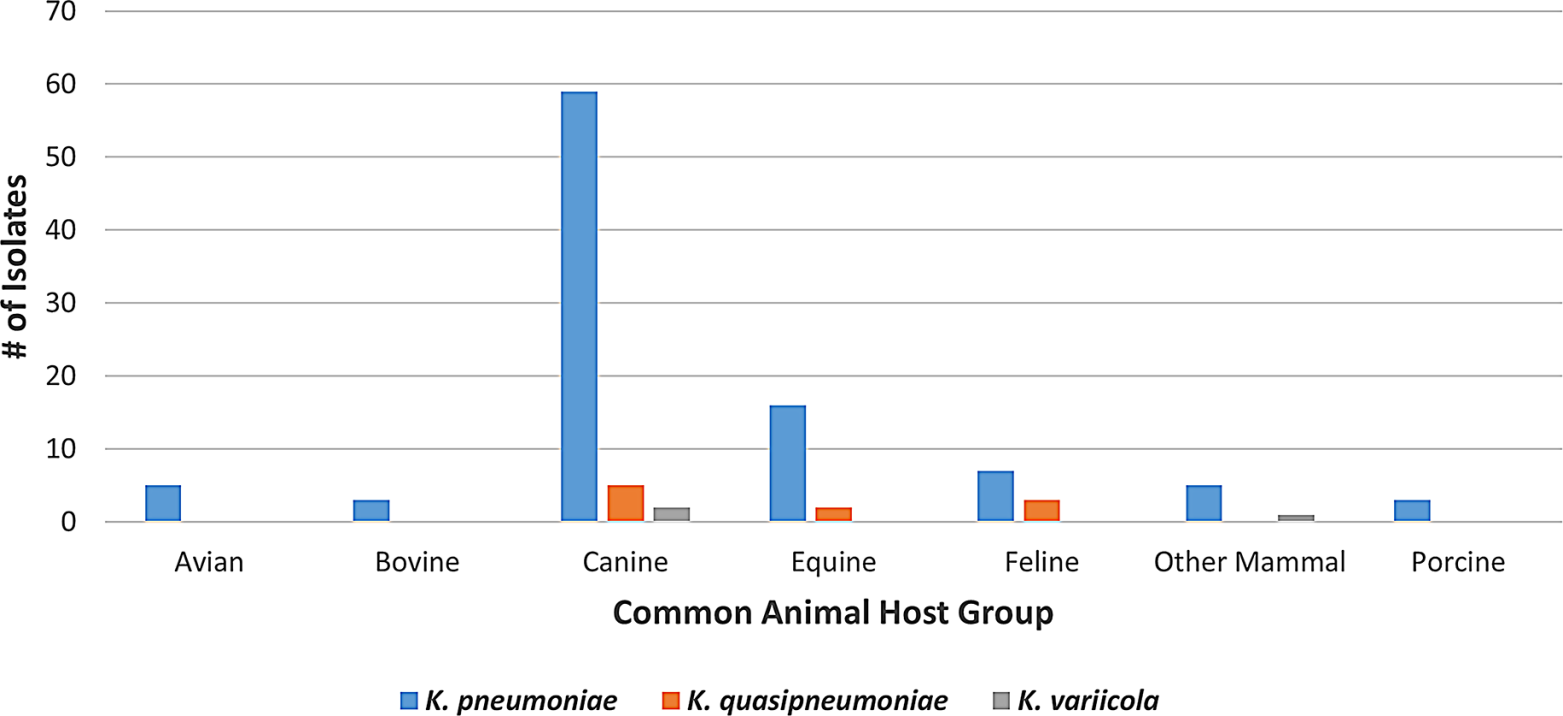
Plasmid-containing isolates by organism and common animal host group. The X axis shows common animal host groups, and the Y axis represents the number of isolates containing plasmids

*K. pneumoniae* isolates that contained plasmids were found in all animal hosts, while *K. quasipneumoniae* was collected only from canines, equines, and felines. *K. variicola* were only isolated from canines and other mammals (Fig. 8).

Only two isolates, OTH-19-VL-LA-TX-0004 and OTH-20-VL-SD-MI-0033, contained multiple copies of a plasmid replicon, and both were Col(IMGS31) found in *K. pneumoniae* isolated from dogs. These were also the only two isolates containing Col(IMGS31) found in the study. Both isolates also contained plasmid IncFIB(K), had identical antimicrobial resistance profiles, were MLST ST307, and were assigned to the same NCBI Pathogen Detection SNP Cluster, PDS000108580.1 (Supplemental File 2), although they were isolated in different years and from different states. OTH-19-VL-LA-TX-0004 was collected in 2019 from Texas and OTH-20-VL-SD-MI-0033 was collected in 2020 from Michigan. The most prevalent plasmid replicons were IncFIB(K) (*n* = 62), found most commonly in isolates from canines (*n* = 41), equines (*n* = 7), and felines (*n* = 5). The next most prevalent plasmids were IncR (*n* = 21), which was most common in isolates from canines (*n* = 10) and equines (*n* = 6), and IncFII(K) (*n* = 20), which was most common in isolates from canines (*n* = 11) and felines (*n* = 4). All other plasmid replicons were represented in fewer than 10 isolates.

## Discussion

*Klebsiella* is an important pathogen of humans and animals, but it has not been sequenced extensively as part of any US national AMR monitoring program. The main goal of this study was to assess the prevalence of antimicrobial resistance genes in our study population (*Klebsiella* isolated from sick animals collected under Vet-LIRN’s AMR Monitoring Program), as well as the relatedness between *Klebsiella* spp. isolated from humans and animals. Most of the isolates in our study originated from clinically ill dogs (67.6%), but there were also clinical isolates from a number of other animal hosts (12.3% equine, 8.3% feline, 4.9% other mammals, 2.9% avian, 2.5% bovine and 1.5% porcine). Similarly, a recent study in Portugal examining *Klebsiella* strains from clinically ill animals revealed that it was most commonly isolated from dogs, in 62% of cases [31]. Within the genus, *K. pneumoniae* is the species most often isolated from ill companion animals [32, 33]. However, more recently, *K. oxytoca* has also been isolated from animals with diverse types of infections [20, 32].

The isolates in our study included multiple *Klebsiella* species, most commonly *K. pneumoniae* (73.0%), followed by *K. quasipneumoniae* (14.7%), *K. variicola* (7.4%), *K. aerogenes* (2.5%), *K. oxytoca* (2.0%), and *K. grimontii* (0.5%). Even though two-thirds of the animal hosts in our study were canine, only one *K. oxytoca* isolate came from a dog, while in a different study conducted in Portugal [31], 22% of *Klebsiella* spp. isolated from dogs were *K. oxytoca*. A study conducted in South Korea in 2021 [20] found that 25% of *Klebsiella* spp. isolated from clinically ill companion animals were *K. oxytoca*, indicating the rise in *K. oxytoca* cases in companion animals in certain geographic areas. In Europe, in a study conducted in 2004 examining the prevalence of *K. oxytoca* in companion animals, only 1% of isolates were identified as *K. oxytoca* [34]. It is estimated that *Klebsiella* spp. cause 8% of all nosocomial bacterial infections in the United States and in Europe [35]. A study conducted in Europe in 2004 reported that the most common *Klebsiella* species giving rise to infections in humans were *K. pneumoniae* and *K. oxytoca* [36]. Studies from United States and Denmark reported that *K. pneumoniae* accounted for 75–86% of all *Klebsiella* species reported in human patients, while *K. oxytoca* accounted for 13–25% [37, 38].

*Klebsiella* isolates in our study were collected from a variety of anatomical sites, with the urinary tract being the most common, comprising just under half of the isolates, followed by the skin and respiratory tract. Similar trends have been observed in other studies, including the recent Portugal study [31].

Sequencing data showed that our isolates were highly diverse. This was demonstrated by MLST analysis, which identified 88 known STs across all 204 *Klebsiella* spp. isolates and all animal hosts. Notably, MLST sequence types ST307, ST11, and ST60 had more identically clustered isolates than any of the other sequence types. However, it is important to note, due to the nature of this study we do not have additional data available to support evidence of any epidemiological links.

Most of the known STs were isolated from dogs (45 total), which is not surprising due to the large number of dog isolates (67.6%). Importantly, few of these STs were previously identified as virulent *K. pneumoniae* clones, those significantly enriched for siderophores and/or *rmpA* genes, compared with the rest of *K. pneumoniae* [39]. These STs included ST36 [40], which has been linked to bacteremia, as well as ST14, ST15 and ST 35, which have been linked to bacteremia and sepsis [41–44]. In addition, we identified four *K. pneumoniae* isolates as ST1, which is closely related to ST258, an extremely drug-resistant, hypervirulent *K. pneumoniae* clone capable of causing severe, untreatable infections in healthy individuals [39, 45, 46]. These four *K. pneumoniae* ST11 isolates were all multidrug resistant; all had identical AMR profiles conferring resistance to aminoglycosides, beta-lactams, rifamycins, phenicols, trimethoprim, fosfomycin, quinolones, macrolides, colistin, sulfonamides, and tetracyclines and contained the IncR plasmid similar to that previously reported [25]. *K. pneumoniae* ST11 isolates also harbored *bla*_DHA_, similar to previously reported isolates [24], and had been assigned to the NCBI Pathogen Detection SNP Cluster PDS000105711.1 (Supplemental File 2). Two isolates came from canine urinary tracts, one from a canine pancreas and one from an equine respiratory tract. *K. quasipneumoniae* collected from a canine urinary tract did not belong to a known sequence type.

Most isolates have not been assigned SNP clusters due to the uniqueness of their sequences, despite over 70,000 *Klebsiella* sequences in NCBI databases. This demonstrates the need for additional sequencing of unique and genetically diverse isolates, especially from animal or environmental sources. Nevertheless, it is reassuring that relatively few animal isolates were closely related to those from humans, despite the One Health nature of this pathogen as it commonly infects both humans and animals. This indicates that animals may not be a common source of human *Klebsiella* infections, similar to previously reported work [47].

Findings related to AMR demonstrated the diversity of resistance genes, including 36 different genes across 11 drug classes and three types of point mutations. Of particular concern was the high prevalence of mechanisms conferring resistance to cephalosporins and fluoroquinolones, two of the most common drug classes used to treat *Klebsiella* infections in animals and humans [48]. The most common resistance classes in our study were beta-lactams (99.0%), followed by phenicol/quinolone resistance due to oqxAB genes (98.5%), and fosfomycin resistance due to fosA genes (97.5%). Among beta-lactams, the most common resistance genes were *bla*_SHV_ (71.2%). Other studies reported high prevalence of oqxAB, fosA and *bla*_SHV_ genes in *K. pneumoniae* isolates from humans and different animal species [49–52]. Fifty-one isolates (25%) also had *gyrA* mutations and/or *qnr* genes, conferring resistance to fluoroquinolones, which in addition to the presence of beta-lactamases, indicates the potential compromised ability to treat animals with infections caused by these bacteria [53, 54]. The presence of multiple additional resistance genes in these isolates could further co-select for these resistant pathogens if animals are exposed to other antimicrobials, such as tetracyclines or sulfonamides.

One of the notable findings from our study was a finding of an isolate with colistin resistance gene, *mcr-8.1* isolated from a goat. Colistin, often termed a last-resort antibiotic [55, 56], is employed when other antibiotics fail, making any form of resistance against it a grave concern. Mobile colistin resistance genes, such as *mcr* variants, pose a significant threat, as they are capable of horizontal transfer between bacteria, thereby potentiating the spread of resistance [57]. The discovery of *mcr-8.1* gene is of concern because it confers resistance to colistin [58]. The isolate had only one plasmid, IncFII(K), which was previously reported as a carrier for *mcr-8.1* gene [59, 60]. The presence of colistin resistance genes, particularly in food animals, is of concern both due to food safety and the potential for environmental spread, including in agriculture. Therefore, it is important to closely monitor the presence and spread of *mcr* genes in animal populations. Such monitoring can help in timely interventions, policy formulation, and strategic planning in both animal husbandry and public health sectors to combat the One Health threat posed by antimicrobial resistance.

While this study did not identify carbapenem resistance genes such as *bla*NDM-5, other studies have reported its presence in animal isolates from *Klebsiella* spp [51, 61, 62]. The presence of *bla*_NDM_ variants in isolates from animals has been a growing concern due to the potential for resistance gene transmission across the animal-human interface [63].

To our knowledge, this is the largest study investigating the genetic diversity of *K. pneumoniae* complex isolates originating from animal clinical isolates in the United States using a genomics-based approach.

## Conclusion

This study demonstrates that animal *K. pneumoniae*-complex infections in the United States are caused by genetically diverse lineages that cluster separately in a majority of cases, with only a few isolates clustering closely to human isolates. This indicates a potential low risk of direct *K. pneumoniae* complex transmission between humans and animals while still highlighting the potential animal health impact and spread of mobile resistance genes to other pathogens of animal and human health concern. Findings from this study highlight the importance of AMR monitoring of veterinary pathogens as a part of the integrated One Health approach.

## Methods

### Participating laboratories and isolate collection

Isolates were collected from 26 states by a network of 30 Vet-LIRN veterinary diagnostic laboratories (“Source laboratories”) from January 2019 to December 2020. All laboratories were affiliated with either an academic institution or the U.S. state department of agriculture. Laboratories were instructed to select only one isolate per client submission. Isolate species were determined by either the analytical profile index (API), matrix-assisted laser desorption/ionization time of flight (MALDI-TOF) mass spectrometry, polymerase chain reaction (PCR), Sensititre, Vitek, or biochemical identification. Species were later confirmed by whole-genome sequencing data (described below).In cases where they differed, whole-genome sequencing was used to make final determination.

Source laboratories submitted metadata for each isolate while anonymizing certain features by omitting specific geographic location and client information. Metadata were collected using the metadata sheet developed by the GenomeTrakr program [64], with additional information needed by the Vet-LIRN Program Office. Those fields included the information on which source lab collected the isolate, Vet-LIRN specific isolate ID, isolate taxonomic name, date of collection (day, month, or year), U.S. state, specific animal host, case type (primary, secondary, tertiary), and the anatomical site from which the pathogen was isolated. A complete metadata sheet template is provided in Supplemental file 6. Out of 208 isolates collected by source laboratories, 207 were sequenced. WGS results showed that three isolates were not *Klebsiella* and were excluded from analysis, leaving 204 *Klebsiella* isolates in the final analysis.

### Genomic DNA extraction and whole-genome sequencing

Purified cultures were grown on plates containing tryptic soy agar supplemented with 5% sheep blood (Remel, Lenexa, KS) and incubated aerobically overnight at 37 °C. Genomic DNA was extracted using the QIAamp 96 DNA QIAcube^®^ HT kit (Qiagen, Germantown, MD, USA) on the Qiagen QIAcube^®^ HT automated liquid handling robot (QIAcube^®^ HT, Qiagen) per the manufacturer’s instructions. DNA was quantified using a Qubit fluorometer (Life Technologies, MD, USA). Sequencing libraries were prepared using Illumina Nextera XT library preparation kits and sequenced using v3 reagent kits 2 × 300 bp chemistry on the Illumina MiSeq platform (Illumina, San Diego, CA, USA).

### Sequence analysis

Sequence analysis was performed using the GalaxyTrakr platform (https://galaxytrakr.org). Paired-end sequence reads were trimmed using Trimmomatic version 0.36.4 [65]. Assembly and MLST sequence typing were performed using skesaMLST version 0.04 [66], which incorporates SKESA for assembly [67], and the PubMLST database [68]. All sequences with coverage below 20X were repeated until they passed this threshold. Culture purity was checked using Kraken version 2.1.1 [69], and any isolate that showed signs of contamination was reisolated for purity and re-sequenced. Isolates that were not Klebsiella were excluded from further analysis. Classification was confirmed using FastANI version 1.3 [70] with matches of 95% and higher considered a positive identification.

Assemblies were screened for AMR genes using AMRFinderPlus version 3.8.28 [71]. AMR plasmids were screened for in the PlasmidFinder database [72]) using the staramr tool version 0.7.2 [73].

All sequencing reads were uploaded to the National Center for Biotechnology Information (NCBI) SRA under BioProject PRJNA325243 and are publicly available at the NCBI Pathogen Detection Browser (https://www.ncbi.nlm.nih.gov/pathogens/isolates#PRJNA325243). Isolate-level accession numbers are listed in Supplemental File 7.

### Phylogeny

To check isolate relatedness, the MiSeq short read contig assemblies were aligned, and a kmer-based SNP analysis was performed using kSNP4 version 4.0 [74]. The optimal kmer length used for alignments was determined to be 19 based on the Kchooser4 tool built into kSNP4. The kSNP4 outputs were processed using the software MEGA11 [75] to create maximum likelihood phylogenetic tree files of the core genome. The interactive Tree of Life version 6.7 [76] was used to construct and visualize phylogenetic trees with surrounding dataset color rings.

## Electronic supplementary material

Below is the link to the electronic supplementary material.

Supplementary Material 1: Supplemental File 1:Animal Hosts and Collection Sites.

Supplementary Material 2: Supplemental File 2: NCBI SNP Clusters.

Supplementary Material 3: Supplemental File 3: MLST Sequence Types.

Supplementary Material 4: Supplemental File 4:AMR Profiles.

Supplementary Material 5: Supplemental File 5: MDR Resistance Classes.

Supplementary Material 6: Supplemental File 6: Metadata Entry Form NCBI.

Supplementary Material 7: Supplemental File 7: NCBI Accession Numbers.

## Data Availability

No datasets were generated or analysed during the current study.

## Abbreviations

AMR: Antimicrobial resistance
API: Analytical profile index
ARGs: Antimicrobial resistance genes
AST: Antibiotic susceptibility testing
CARB: Combating Antibiotic-Resistant Bacteria
GLASS: Global Antimicrobial Resistance and Use Surveillance System
NAHLNCVM: Center for Veterinary Medicine
FDA: Food and Drug Administration
MALDI-TOF: Matrix Assisted Laser Desorption/Ionization-Time of Flight
MLST: Multilocus Sequence Typing
NARMS: National Antimicrobial Resistance Monitoring System
NCBI: National Center for Biotechnology Information
PCR: Polymerase chain reaction
SNPs: Single nucleotide polymorphisms
USDA: United States Department of Agriculture
Vet-LIRN: Veterinary Laboratory Investigation and Response Network
WGS: Whole-genome sequencing
